# Heterotopic respiratory mucosa in the scalp overlying abnormal bony island in the skull linked to maternal misoprostol use, literature review and surgical experience

**DOI:** 10.1016/j.ijscr.2019.03.010

**Published:** 2019-05-09

**Authors:** Taghreed Alhumsi, Malak Alghamdi, Ikhlas Altowaijri, Abdullah Alqahtani, Ahmed Alhumidi

**Affiliations:** a Plastic Surgery Department King Saud University, King Saud University Medical City, Riyadh, Saudi Arabia; b Department of Pediatrics, King Saud University, King Saud University Medical City, Riyadh, Saudi Arabia; c Neurosurgery Department, King Saud University, King Saud University Medical City, Riyadh, Saudi Arabia; d Prince Sattam Bin Abdulaziz University, College of Medicine, Alkharij, Saudi Arabia; e Department of Pathology, College of Medicine, King Saud University, Riyadh, Saudi Arabia

**Keywords:** Case report, Misoprostol, Heterotopic respiratory, Skin tag, Skull bony island

## Abstract

**Background:**

Congenital scalp lesions rarely occur in children. These lesions are usually noticed at birth and mostly comprise skin and bone tissue loss in the form of acutis aplasia congenita. The management of these lesions is highly dependent on their location and the affected underlying structures.

**Case report:**

We report the case of a baby girl who was referred to us after a normal full-term delivery and who had an area of an abnormal overgrowth of skin (skin tag) on the vertex of the scalp, with an area of surrounding alopecia. She was otherwise healthy with no noted congenital anomalies. Apart from a prenatal history of attempted abortion using misoprostol, she had no significant history or congenital anomalies. She underwent a CT scan and an MRI. She also underwent an operation to excise the overgrowth and to address the underlying bone tissue anomaly. Histopathology showed respiratory mucosa heterotopia. We report this unusual case that suggests the need for an algorithm to manage such cases, as well as the need to investigate the possibility of misoprostol teratogenicity and a probable link to heterotopia.

**Conclusion:**

Congenital skin lesions on the scalp should be approached in a multidisciplinary manner to guide their treatment.

## Highlights

• We present a case of abnormal skin overgrowth over an abnormal bony island in a full tern child after misoprostol use.
• Central vertex lesions can vary from overgrowth and undergrowth or under formation of tissues.
• Careful assessment of any child with skull lesion.
• Management algorithm proposes management steps that we applied in our case and can help manage other cases of scalp lesions in pediatric patients.

## 1 Introduction

Skin tags (fibroepithelial polyps, or acrochordons) are terms used to describe benign skin lesions, which consist of tissue projecting from the surrounding skin [1]. The incidence of acrochordons in newborns in some cross-sectional studies was reported to be approximately 0.6%, which comprised accessory tragus (preauricular tag) [2]. We have found no reports of central scalp skin tags in the literature. However, congenital midline scalp lesions and defects are not uncommon occurrences and have been reported since 1826 [3]. Such conditions include aplasia cutis congenita (ACC), vertex encephaloceles (VC), and other conditions that are usually accompanied by underlying bony, dural, or vascular anomalies [4].

Misoprostol is a synthetic prostaglandin E1 analogue used for treatment and prevention of peptic ulcers induced by NSAIDs. It is also used in the first trimester to stimulate uterine contractions, either alone or in combination with other medications, for nonsurgical abortion [5]. However, its failure has been linked to many congenital anomalies. These may include limb defects, syndactyly, club foot, Poland sequence, and encephalocele [6].

Heterotopia is defined as a mass of normal tissue in an abnormal location [7]. Many types of heterotopia have been reported in different locations in the body [7]. In the head and neck, the most common types of heterotopia are nasal glial heterotopia (nasal glioma) and cutaneous heterotopic meningeal nodules (primary cutaneous meningioma) [8].

Herein, we present an abnormal case of a central scalp lesion with underlying abnormal bony island in the area and bony protrusion linked to maternal history of misoprostol usage which was managed in a university hospital and it was written according to SCARE criteria [9]. Further surgical assessment and approaches are discussed.

## 2 Case report

**Fig. 1:**
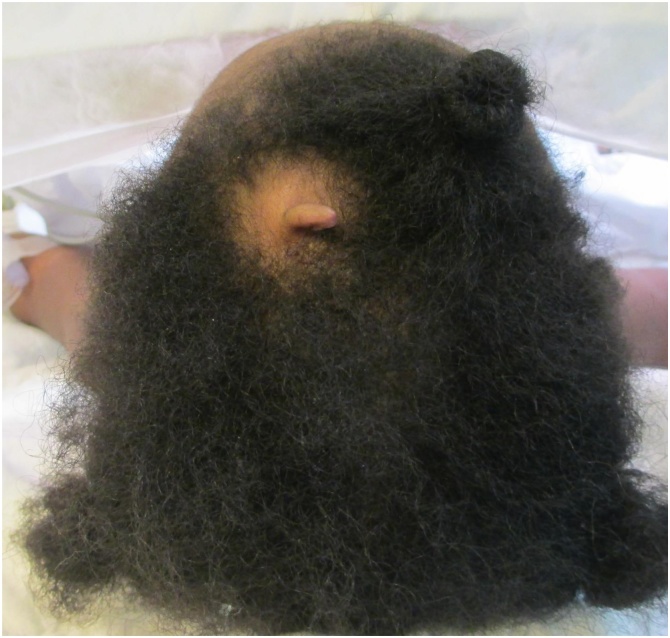
Clinical picture showing tissue outgrowth and area of alopecia on scalp vertex. (Pre-operative photo shown scalp skin tag).

A newborn female was referred to us after a normal full-term delivery with an area of an abnormal overgrowth of skin (skin tag) on the vertex of the scalp and surrounding alopecia. She was otherwise healthy with no noted congenital anomalies (Fig. 1).

She was born to nonconsanguineous parents and was the fourth in the family with no similar history or history of other congenital anomalies; there was no history of miscarriage in the family.

In the prenatal history, it was discovered that her mother, a 32-year-old healthy woman, G5, P3, attempted to terminate the pregnancy with the use of misoprostol orally, vaginally and sublingually at the fourth week of gestation. The pregnancy was not terminated. All prenatal follow-ups thereafter showed a healthy child with no concerns during pregnancy and normal fetal ultrasound throughout all trimesters.

**Fig. 2:**
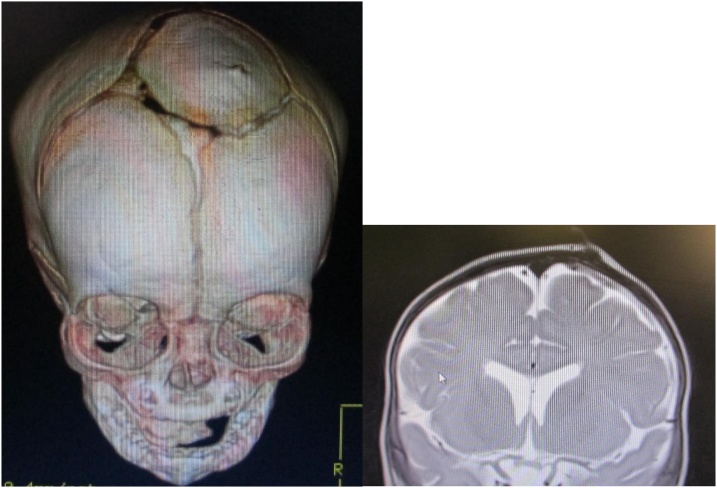
3D computed tomography and magnetic resonance imaging showing abnormal bony island and connecting vessel. MRI showed a soft tissue skin tag in the upper left frontal parasagittal region, with a tiny vascular connection to the intracranial cortical vein over the brain surface 2–3 mm from the sagittal sinus. (On the left, 3D-CT of skull shown a small central bony spur corresponding to the skin tag area. On the right, MRI showed a soft tissue skin tag in the upper left frontal parasagittal region, with a tiny vascular connection to the intracranial cortical vein over the brain surface 2–3 mm from the sagittal sinus).

The patient was reviewed in the plastic surgery and neurosurgery clinics. She was investigated with CT scan with 3D reconstruction to delineate any bony abnormalities. CT scan showed patent sagittal, coronal and lambdoid sutures, with an abnormally located vertex bony island in the area of anterior fontanel and parasagittal sinus; there was a small central bony spicule corresponding to the skin tag area (Fig. 2). She was further investigated by MRI, which showed a soft tissue skin tag in the upper left frontal parasagittal region, with a tiny vascular connection to the intracranial cortical vein over the brain surface 2–3 mm from the sagittal sinus(Fig. 2).

In light of her clinical and radiological findings, excision of the skin tag with surrounding alopecia was planned at the age of 1 year; this included excision of the underlying bony spicule, either by burr or craniotomy, if necessary, to control bleeding at the base of the bony spicule, due to the proximity of the feeding vessel to the sagittal sinus. In anticipation of an extended recovery, a pediatric intensive care unit bed was booked.

**Fig. 3:**
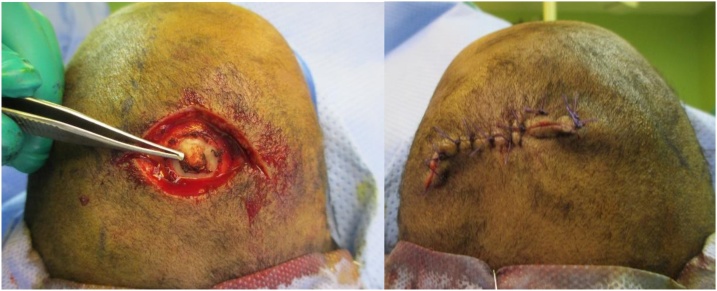
Intraoperative and postoperative images. (On the left, Intra-operative photo shown the defect and bony spur. On the right, post-operative photo shown the primary closure).

A multidisciplinary team was involved in the procedure, including both plastic surgery and neurosurgery teams. A zig-zag incision was marked with a diamond excision around the area of alopecia. The area was excised down to the pericranium, and the underlying bony spicule was burred down to a level where the cranium was smooth. Hemostasis was secured, and no major bleeding was encountered intraoperatively. Closure was done after minor mobilization of local tissues with no tension. (Fig. 3).

**Fig. 4:**
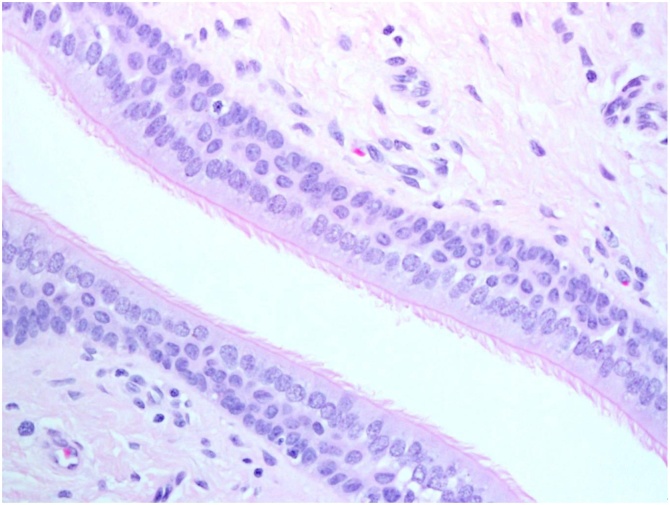
Low power view of the scalp lesion showing a small cyst of respiratory mucosa on the right side of the image and benign cartilage tissue on the left side (H/E stain, original magnification ×20). (Heterotopic respiratory mucosa).

Histopathologic assessment of the excised tissue showed benign polypoidal skin with underlying fibrocartilaginous tissue and heterotopic respiratory mucosa (Fig. 4).

The patient showed uneventful postoperative recovery. The baby was followed up for 12 months; there were no complications, and she was developmentally appropriate for her age and thriving well. The family consented to the publication of this case.

## 3 Discussion

Congenital midline scalp lesions and defects are not uncommon occurrences and have been reported since 1826 [3]. Such cases have to be managed with care. Preoperative evaluation, including clinical history and neuroimaging studies, will help to generate a differential diagnosis and exact anatomic localization of the lesion [10]. The differential diagnosis is broad, and radiologic evaluation is often requested.

Aplasia cutis congenita (ACC) is a skin condition that occurs in newborns. It is a disease of localized or widespread complete or partial loss of skin [11]. It may also involve subcutaneous tissue, bone, and/or dura [4]. Causes include placental infarcts, genetics, teratogenic substances, intrauterine infections and trauma, ectodermal dysplasia, imperfect neural tube closure and maternal intrapartum drug use [11]. While it has been reported in other areas of the body such as the trunk by Singh et al. [12], 70% of cases were found on the scalp, especially in the vertex area. Lesions usually present with no hair, apart from some sparse hairs that may grow in the area at later times [13]. In regards to causes of ACC, vascular anomalies have been implicated in its etiology, and ACC has been reported to be associated with superficial dilated veins communicating with the sagittal sinus [14]. ACC has been associated with cutaneous meningeal heterotropia, as an incidental finding on excision of an old alopecic scar after conservative management and healing [15]. Treatment of ACC depends on the severity of the condition and whether it is associated with other congenital anomalies. Treatment options range from conservative management for small lesions to operative treatment for larger lesions [11]. Another type of midline lesion is vertex encephaloceles (VC), which is also known as midline interparietal cephaloceles; it presents as skin-covered subscalp lesions with extracranial meningeal and/or neurological tissue, with an underlying bony defect. These lesions are frequently associated with venous anomalies. The most common of these anomalies is the vertical embryonic positioned straight sinus [16]. VC lesions also present with a surrounding area of atrophic alopecia, with an area of coarse hair on the edges (“hair collar sign”) or a vascular lesion, such as skin angioma, surrounding the center of the lesion [17]. The treatment of such conditions comprises surgical excision of the lesion and closure with a watertight seal of the dura [18]. Scalp cysts located in areas other than the midline or vertex may also present with brain heterotropia, despite normal underlying skull bone. Pryce et al. [19] reported a hairless multilocular skin lesion overlying the left parietal bone in a 3-month-old child. Histopathological examination of the excised lesion showed ectopic glial cells. If associated with other anomalies, such as skull bone defects and surrounding dura, the abnormal glial tissue is a variant of encephalomeningocele [20].

In our case, we encountered an overgrowth of skin, presenting as a skin tag rather than skin loss. The area surrounding the tag had complete alopecia, and the underlying tissue showed an abnormal bone, which seemed like an excess bony island, corresponding to the area of the anterior fontanelle. This abnormal bone had a central spicule corresponding to the area of the skin outgrowth, with an underlying vessel connecting it to the sagittal sinus. Notably, our patient’s presentation was the opposite of most scalp lesions, with most elements in excess, rather than a loss of tissue. The heterotopia encountered in our case was of respiratory mucosa and not brain tissue, as described in previous reports of ACC or other scalp lesions.

Misoprostol is a synthetic prostaglandin E1 analogue used for treatment and prevention of peptic ulcers induced by NSAIDs. It is also used in the first trimester to stimulate uterine contractions, either alone or in combination with other medications for nonsurgical abortion [5]. However, many congenital malformations have been reported after failure to terminate pregnancies by using misoprostol in early pregnancy, and it has been labeled as a teratogenic drug [6]. Teratogenic factors affect both embryos and fetuses between fertilization and birth. The fetus is less sensitive to morphological alterations than the embryo; however, changes in functional capacity, intellect, reproduction or renal function may occur. Teratogenicity depends on the gestational age at the time of exposure. Exposure as early as two weeks will destroy all cells and result in spontaneous abortion; however, exposure in the embryonic phase (up to eight weeks) will result in a major malformation. Later exposure in the fetal phase (from nine to thirty-eight weeks) causes changes in the functional capacity, intellect, or reproductive ability [21]. In regards to misoprostol, due to contractions caused by the drug, a transient drop in fetal circulation occurs, affecting the intensity of vascular supply, which can cause most of the congenital anomalies [6]. Cavalcante et al. [22] reported the cases of 15 mothers of children diagnosed with Moebius Syndrome; 66.7% of these women attempted abortion with misoprostol. Uterine contractions caused by misoprostol can induce hemorrhage and cell death of cranial nerves as a result of embryo flexion, which causes Möbius syndrome. It may also disturb blood flow, leading to hypoxemia and ischemia, which can cause limb defects, syndactyly, club foot, Poland sequence, and encephalocele [6]. Our patient presented with a positive prenatal history of attempted abortion with misoprostol during the fourth week of pregnancy. However, we have found no evidence linking misoprostol to scalp or vertex skin or skull anomalies or lesions, nor to heterotopia, in the literature. Other anomalies associated with misoprostol use did not occur in our patient, and she is currently thriving with no associated congenital anomalies.

Heterotopia is defined as the presence of histologically normal tissue in an ectopic anatomic location [7]. It may occur anywhere in the body, as tissue ectopic to the anatomical location. In the head and neck, the most common types of heterotopia are nasal glial heterotopia (nasal glioma) and cutaneous heterotopic meningeal nodules (primary cutaneous meningioma).^(8)^ Brain cell heterotropia is reported as one of the major anomalies in the brain, causing epilepsy and neurodevelopmental issues. Its causes include genetic factors, such as periventricular heterotopia, as well as environmental factors, such as teratogens. Environmental factors causing neurological heterotopia have been demonstrated on post-mortem brain autopsies in children who were exposed to the substances prenatally [23]. However, respiratory epithelium heterotopia has been reported in the tongue, mandible, eyes, rectum, thyroid, skin and uterus [24]. In the orbit, respiratory heterotopia is reported in half of the cases, due to orbital trauma and surgical fracture reconstruction [25]. In our case, we encountered respiratory mucosal heterotopia in an abnormal skin tag over an abnormal excess bone, which arguably is not a defect in which tissues can sequestrate. To our knowledge, this is the first case of respiratory mucosa heterotopia in a skin tag in the scalp, accompanied by alopecia and abnormal excess bone with bony spicule.

**Fig. 5:**
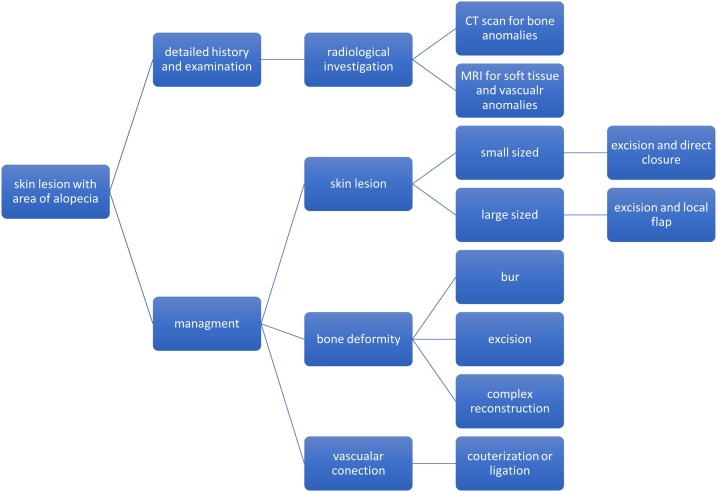
a proposed algorithm for the management.

Management of congenital scalp vertex lesions is challenging and requires a multidisciplinary approach, usually involving a craniofacial surgeon, neurosurgeon, geneticist, and histopathologist. In our case, we started with a thorough history. Detailed prenatal, antenatal and postnatal history is essential for guiding management of the case. A history of consanguinity and genetic history should also be obtained. Thorough examination of the child and exclusion of other anomalies is necessary. Radiological investigations are key to delineate the extent of the deformity. In our case, we carried out CT scan with 3D reconstruction to visualize the bony anomaly, and used MRI to visualize vascular anomalies. We suggest a proposed algorithm to manage such cases in the future, where elements of both skin and bony anomalies should be addressed, along with any vascular anomalies, if present. In our algorithm, we suggest managing each part of the deformity as follows: first, skin excess should be managed for excision and closure, with a primary or local flap. Second, bony deformity should be managed in accordance with the need for excision, burr, or more complex reconstructive options. We believe that a stepwise approach will help manage such cases with ease and help patient recovery post-operatively. The algorithm is shown in Fig. 5.

## 4 Conclusion

We have reported an unusual case involving a central scalp skin tag in a full-term child after failed abortion using misoprostol. Careful evaluation of such cases is needed to diagnose and differentiate them from ACC or VC, which are the two most common congenital skin lesions on the vertex. Managing such cases should involve careful consideration of the location of the lesion, as well as the possibility of affecting underlying structures during surgical management. Our algorithm proposes management steps that we applied in our case and can help manage other cases of scalp lesions in pediatric patients.

Misoprostol has been implicated as a cause of many congenital anomalies. To our knowledge, it central scalp skin tag with heterotopia has not been reported as an effect of misoprostol teratogenicity. Further investigation is necessary to verify that misoprostol was the cause of the condition.

## Conflicts of interest

No conflicts of interest.

## Sources of funding

We don’t have any source of funding.

## Ethical approval

The study is exempt from ethnical approval.

## Consent

Written informed consent was obtained from the patient for publication of this case report and accompanying images and it is attatched.

## Author contribution

1) **Taghreed Alhumsi** was involved in data collection and overall manuscript writing.
2) **Malak Alghamdi** was involved in genetic part of manuscript writing.
3) **Ikhlas Altowaijri** was involved in neurosurgical part of manuscript writing.
4) **Abdullah Alqahtani** was involved in the overall manuscript organization.
5) **Ahmed Alhumidi** was involved in the histology data reporting and figures assembly.

## Registration of research studies

researchregistry4562.

## Guarantor

Abdullah Alqahtani.

## Provenance and peer review

Not commissioned, externally peer reviewed.
